# Lipid metabolism in *Calanus finmarchicus* is sensitive to variations in predation risk and food availability

**DOI:** 10.1038/s41598-020-79165-6

**Published:** 2020-12-18

**Authors:** Elise Skottene, Ann M. Tarrant, Dag Altin, Rolf Erik Olsen, Marvin Choquet, Kristina Ø. Kvile

**Affiliations:** 1 Department of Biology, Norwegian University of Science and Technology, Trondheim, Norway; 2 Department of Biology, Woods Hole Oceanographic Institution, Woods Hole, MA USA; 3 BioTrix, Trondheim, Norway; 4 Faculty of Biosciences and Aquaculture, Nord University, Bodø, Norway; 5 Centre for Ecological and Evolutionary Synthesis (CEES), Department of Biosciences, University of Oslo, Oslo, Norway; 6 Norwegian Institute for Water Research, Oslo, Norway

**Keywords:** Computational biology and bioinformatics, Developmental biology

## Abstract

Late developmental stages of the marine copepods in the genus *Calanus* can spend extended periods in a dormant stage (diapause) that is preceded by the accumulation of large lipid stores. We assessed how lipid metabolism during development from the C4 stage to adult is altered in response to predation risk and varying food availability, to ultimately understand more of the metabolic processes during development in *Calanus* copepods. We used RNA sequencing to assess if perceived predation risk in combination with varied food availability affects expression of genes associated with lipid metabolism and diapause preparation in *C. finmarchicus*. The lipid metabolism response to predation risk differed depending on food availability, time and life stage. Predation risk caused upregulation of lipid catabolism with high food, and downregulation with low food. Under low food conditions, predation risk disrupted lipid accumulation. The copepods showed no clear signs of diapause preparation, supporting earlier observations of the importance of multiple environmental cues in inducing diapause in *C. finmarchicus*. This study demonstrates that lipid metabolism is a sensitive endpoint for the interacting environmental effects of predation pressure and food availability. As diapause may be controlled by lipid accumulation, our findings may contribute towards understanding processes that can ultimately influence diapause timing.

## Introduction

Calanoid copepods are important zooplankton species in the North Atlantic and Arctic marine ecosystems. These calanoid copepods convert carbon from phyto- and microzooplankton to accessible energy in the form of wax ester (WE) lipids for higher trophic levels, including commercially important fish stocks^1,2^. *Calanus finmarchicus* (Gunnerus, 1770) typically dominates in subarctic Atlantic areas, e.g. in the Norwegian Sea and the southwestern Barents Sea, while the larger and more lipid-rich *C. glacialis* and *C. hyperboreus* dominate in Arctic waters^3^. In response to the high seasonality in primary production at high latitudes, *Calanus* spp. conduct ontogenetic seasonal vertical migrations. The copepods reproduce near the surface, where eggs are hatched, after which the larvae develop through six naupliar stages (N1-N6) followed by five copepodite (C1–C5) stages. Typically during the last copepodite stage (C5), *C. finmarchicus* either molt directly into the adult stage (C6), or enter a dormant phase, termed diapause, which can last for several months^4–6^. The copepods spend diapause at depth, presumably without feeding, while slowly developing towards adulthood. They rely solely on endogenous energy stores, primarily WE lipids retained within a specialized organ called the lipid sac^7^.

The timing of seasonal vertical migrations likely evolved to maximize fitness of copepods in response to the seasonality of environmental conditions^8^. In freshwater zooplankton, external cues like photoperiod, food availability and predator kairomones have been linked to diapause induction or termination (summarized by Gyllstrøm and Hansson, 2004^9^). However, previous studies have been unable to define explicit links between environmental cues and diapause induction in *C. finmarchicus*, though it has been acknowledged that a combination of several environmental factors are involved, rather than a single cue^8,10^. While most studies have focused on cues like photoperiod and sea surface temperature^10,11^, predation pressure might be a critical and underappreciated environmental driver behind seasonal migrations of this species^8^. In general, predation risk is known to influence a range of life history traits in marine copepods, including growth, development and reproduction^12–14^, but the role of predation risk in shaping zooplankton life history has been much less explored in the ocean than in freshwater and estuarine environments^15,16^.

Regardless of external cues, the ability to accumulate sufficient lipids to survive months without feeding is an important aspect of diapause preparation^17–22^. The Lipid Accumulation Window (LAW) hypothesis postulates that individual copepods must achieve a certain level of stored lipids in order to initiate diapause^22–25^. For *C. finmarchicus,* 70 µg C of WE has been suggested as the minimum threshold for diapause entry^22^. Thus, adequate food availability during the growth season is a prerequisite for diapause initiation to occur. In order to accumulate sufficient lipid levels, lipid biosynthesis must exceed catabolism of lipid stores. Throughout their development, *Calanus* spp. experience dramatic changes in the amount of stored lipids and in the expression of genes associated with lipid metabolism^20,21,26,27^. In pre-reproductive copepods, lipid catabolism can be expected to be low when sufficient food is available, but may increase due to stress^28^ and insufficient food availability^29^. However, downregulation of lipid catabolism by crustaceans in the presence of stressors has also been reported^30–32^, illustrating that lipid metabolism is sensitive to external factors. Studying lipid metabolism on a gene expression level can provide detailed knowledge on how lipid accumulation and catabolism is altered in response to environmental cues, possibly before these changes become evident on a physiological level. Understanding the metabolic processes during late development in *C. finmarchicus*, including those that potentially lead to diapause, can contribute to a better understanding of the energy flow within the marine ecosystem and, potentially, of diapause induction in *Calanus* copepods.

**Figure 1:**
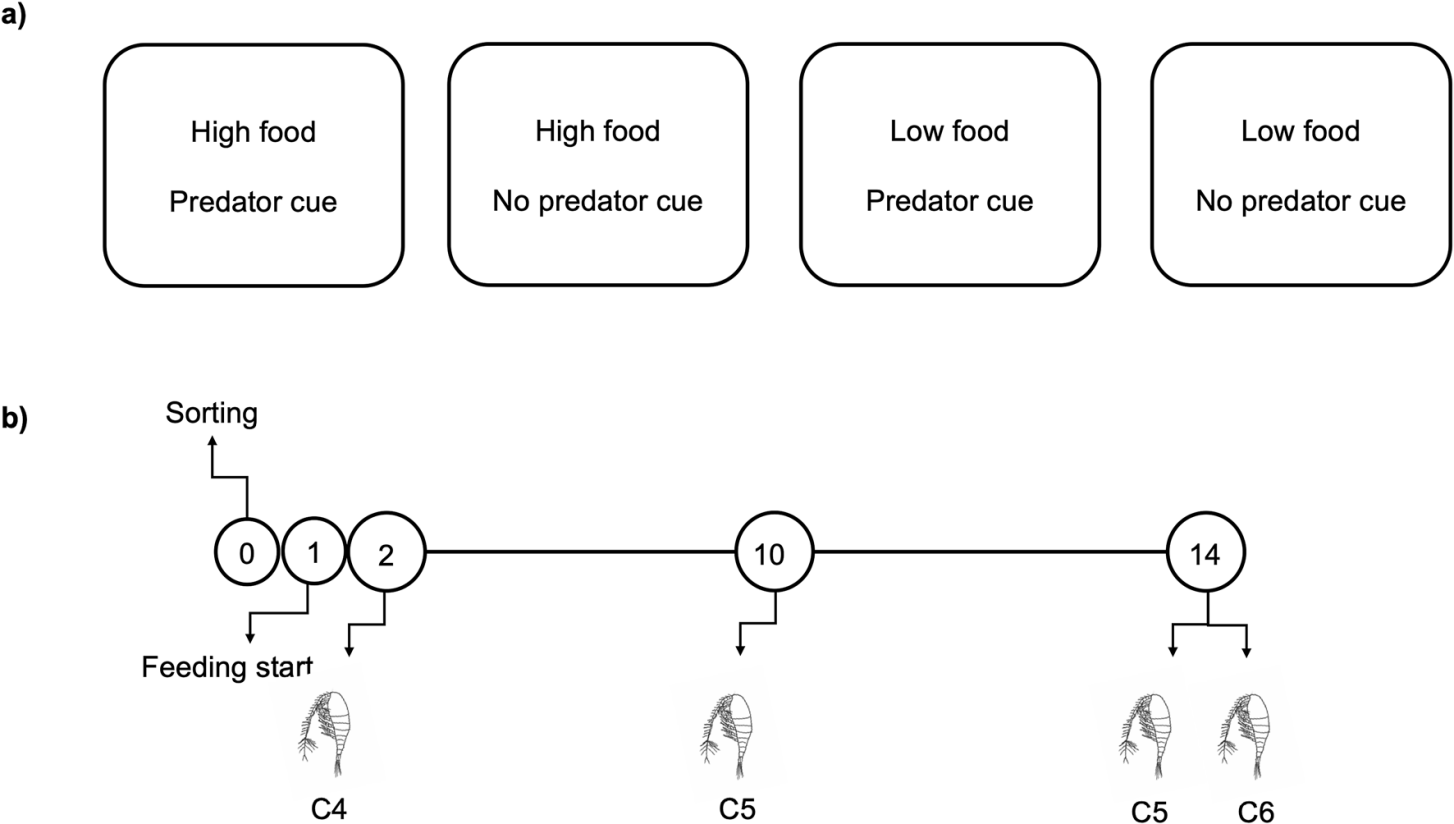
Treatment combinations and timeline of the experiment. (**a**) High food (*Rhodomonas baltica*): 200 μg C/L. Low food: 90 μg C/L. The tanks with predator cue continuously received water from a tank of lumpfish (*Cyclopterus lumpus*) juveniles fed *Calanus finmarchicus*. No predator cue: filtered sea water. Each treatment had three replicate tanks, each of which were sampled on each sampling day. (**b**) Sampling of copepods for RNA-seq was performed on days 2 (stage C4, one tank per treatment only, see “Methods”), 10 (stage C5) and 14 (stages C5 and C6, females). Number of copepods per tank (9 on average) are reported in Supplementary Table 7.

In order to assess this aim, we investigated the effects of perceived predation risk and varying food availability on molecular indicators of lipid metabolism in *C. finmarchicus* copepodites during the transitions from the C4 to the C6 stage (Fig. 1 shows treatment combinations and timeline of the experiment). In a recent study by our group, predation risk led to faster development under high food conditions in *C. finmarchicus*, and the copepods molted into the adult stage (C6) without entering diapause^33^. From these and other previous observations^6,33,34^, we did not expect diapause induction to occur under laboratory conditions. However, as perceived predation risk may serve as a cue for diapause induction, we here also assessed whether the copepods in any of the treatments exhibited changes in gene expression consistent with preparation for diapause. If so, we expected to be able to link this to differences in lipid metabolism. Through RNA sequencing, we assessed expression of genes related to lipid accumulation and catabolism and diapause in the different treatments (Table 1). In this molecular analysis we used a subset of the same copepods from our previous study^33^, from which we re-analyzed lipid content and stage progression data in order to specifically link these measurements with the gene expression results. This was done to provide broader physiological context for the molecular results. The experiment was conducted using *C. finmarchicus* from the NTNU Sealab culture^35^. *Calanus* spp. copepods collected during diapause in the Trondheimsfjord, Norway, were used as a reference group representing a state of diapause and very low metabolism^21^.

**Table 1:** Target genes involved in lipid metabolism/diapause/development selected for differential expression analyses (GLM) in *Calanus finmarchicus* exposed to a combination of presence or absence of a predator cue with high or low food availability.

Abbreviation	Gene name	Function	References
*hsp22*	Heat shock protein 22	Diapause marker	^20,21^
*Ferritin*	Ferritin	Diapause marker	^20,21^
*FABP*	Fatty acid binding protein	Lipid storage/biosynthesis	^20^
*ELOV*	Elongase	Lipid storage/biosynthesis	^20^
Lipid storage genes	Set of lipid storage genes	Lipid biosynthesis	^27,51^
ß-oxidation genes	ß-oxidation genes (number of identified transcripts = 63)	Lipid catabolism	^21,21^
*Torso-like*	Torso-like	Developmental marker	^20,26^

## Results

### Lipid accumulation genes

**Figure 2:**
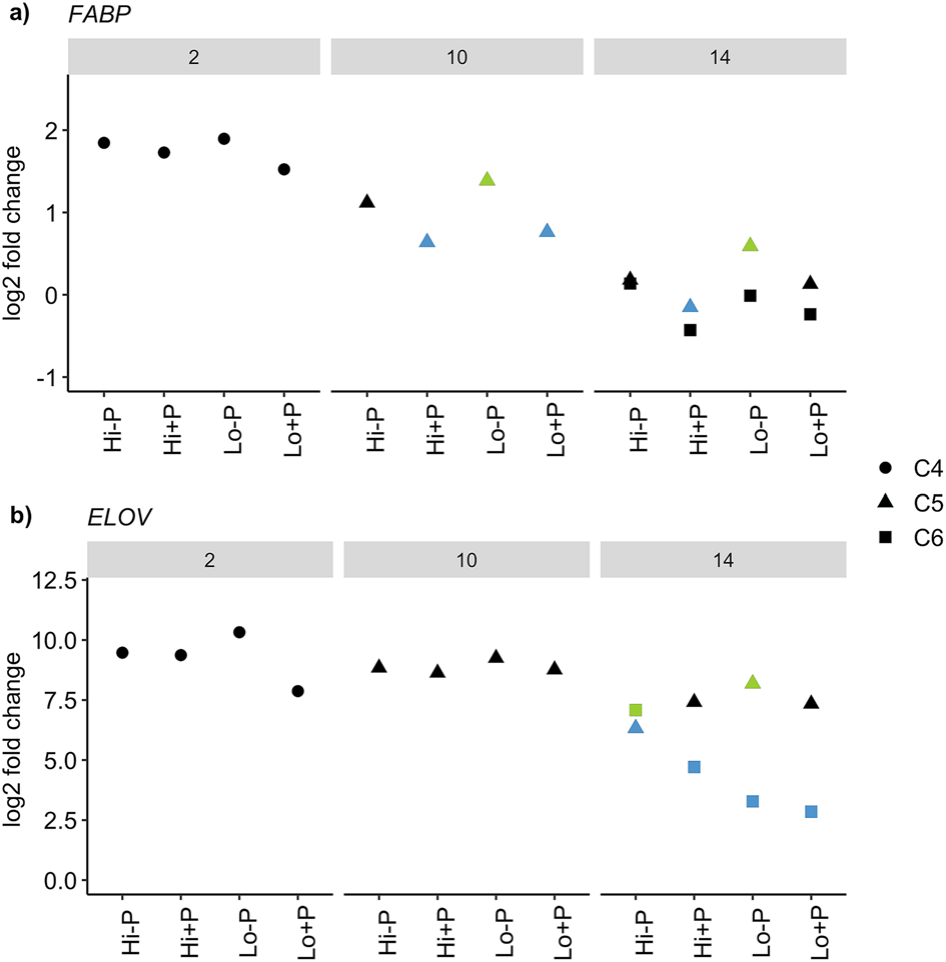
Gene expression of (**a**) fatty acid binding protein (*FABP*) and (**b**) fatty acid elongase (*ELOV*) in *Calanus finmarchicus* copepods exposed to a combination of presence or absence of a predator cue with high or low food availability, compared to the reference group (*Calanus* spp. C5 copepodites in diapause). Bar at the top indicates the number of days between experimental initiation and sampling. Within each day and stage, green symbols denote significant (P < 0.05) upregulation from blue symbols between treatments. Shapes indicate developmental stages (see legend). X-axis shows treatments: *Hi-P *High food and no predator cue, *Hi + P *High food and predator cue, *Lo-P *Low food and no predator cue, *Lo + P *Low food and predator cue. Y-axis shows log2 fold change (FC) relative to the reference group. Not marked in figure: in C6, ELOV was significantly upregulated in Hi + P relative to Lo + P (details in text).

“*FABP*”, a fatty acid binding protein, and “*ELOV*”, which catalyzes elongation of very long chain fatty acids, are known to be upregulated during periods of rapid lipid synthesis/storage in *C. finmarchicus*^20,26^. Both genes were generally upregulated relative to the reference group (*Calanus* spp. C5s collected in the field during diapause), and they showed an overall decline throughout the experiment as development progressed (Fig. 2). Relative to the reference group, *FABP* was upregulated in C4s and C5s from day 10 (henceforth called “early C5s”) in all treatments (Fig. 2a, P < 0.05, all results from statistical tests are available in Supplementary Table 1), but it was not significantly different in C5s from day 14 (called “late C5s”) or C6s. In early C5s, *FABP* was significantly upregulated in the treatment with low food and no predator cue compared to the treatments with a predator cue and high food [log2 fold change (FC) = 0.75, P = 0.02, false discovery rate (FDR) = 0.65], and low food (logFC = 0.63, P = 0.048, FDR = 1, details for all comparisons in Supplementary Table 2). In late C5s, *FABP* was significantly upregulated in the treatment with low food and no predator cue compared with those from high food and predator cue (logFC = 0.75, P = 0.02, FDR = 0.30, Fig. 2a).

*ELOV* was significantly upregulated in all treatment groups on all sampling days compared to the reference group (P < 0.05, Fig. 2b, details in Supplementary Table 1). In C4s, *ELOV* was almost 6 times more highly expressed in the group with low food and no predator cue than the group with low food and predator cue, but the comparison was not significant at the 0.05 alpha level (logFC = 2.45, P = 0.05, FDR = 0.59, Fig. 2b). In late C5s, *ELOV* was upregulated in the group with low food and no predator cue compared to the group with high food and no predator cue (logFC = 1.86, P = 0.02, FDR = 1, Fig. 2b). In C6s, *ELOV* expression was upregulated in the group with high food and no predator cue compared to all other treatment groups (P < 0.05, Supplementary Table 2). ELOV was also significantly upregulated in the treatment with high food and a predator cue compared to those in with low food and a predator cue (logFC = 1.86, P = 0.02, FDR = 1).

Among the suite of genes involved in lipid synthesis identified by Lenz et al*.* (2014) and Tarrant et al*.* (2016), most desaturases, fatty acid synthetases, elongases and phospholipid acyltransferases were found to be strongly upregulated in all copepods in the experiment compared to the reference group, regardless of treatment (Supplementary Table 3).

### Lipid catabolism genes

Catabolism of fatty acids occurs primarily in mitochondria through the ß-oxidation pathway^36^, and 63 gene transcripts (henceforth called “genes") encoding the enzymes in this pathway were recently identified in the *C. finmarchicus* transcriptome^21,21^. We assessed catabolism of lipids by investigating expression patterns of these genes, an approach previously used in studies on *Calanus* copepods^21,32^. To facilitate interpretation, the ß-oxidation gene expression results are referred to as patterns of either up- or downregulation based on the number of genes that are differentially expressed between treatments. For example, one downregulated gene and ten upregulated genes in a comparison indicates a pattern of upregulation.

**Figure 3:**
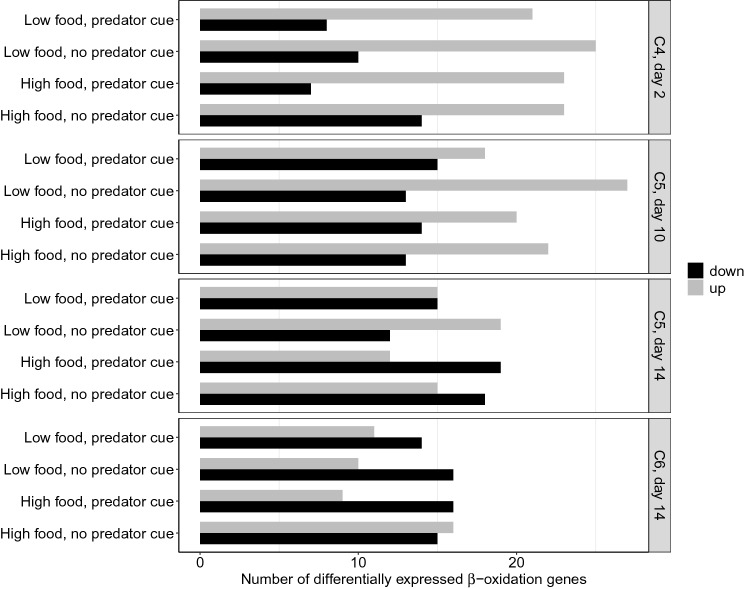
Number of significant (P < 0.05) differentially expressed ß-oxidation genes (x-axis) in *Calanus finmarchicus* in the experimental treatments (y-axis, left) each sampling day (y-axis, right) compared with the reference group (*Calanus* spp. C5 copepodites in diapause). Number of upregulated genes declined with time (linear regression, P = 0.005, F_6,9_ = 11.27, adjusted R^2^ = 0.80). Stages (C4, C5 and C6) were assessed separately. Grey bars indicate upregulated genes and black bars indicate downregulated genes relative to the reference group. One gene upregulated in e.g. Lo + P also implies that the same one gene is correspondingly downregulated in the reference group.

Across treatments, compared to the reference group, there was a general pattern of upregulation of ß-oxidation genes early in the experiment that decreased with time and development [mean number of genes differentially expressed (P < 0.05) across treatments: C4: 23 up, 10 down; early C5: 22 up, 14 down; late C5: 15 up, 16 down; C6s: 12 up, 15 down; Fig. 3, details presented in Supplementary Table 4]. The number of upregulated genes significantly declined with time (linear regression: − 6.50 ± 1.74, P = 0.005, F_6,9_ = 11.27, adjusted R^2^ = 0.80), but there was no corresponding change in the number of downregulated genes (linear regression: 2.25 ± 1.69, P = 0.22, F_6,9_ = 3.08, adjusted R^2^ = 0.45).

**Figure 4:**
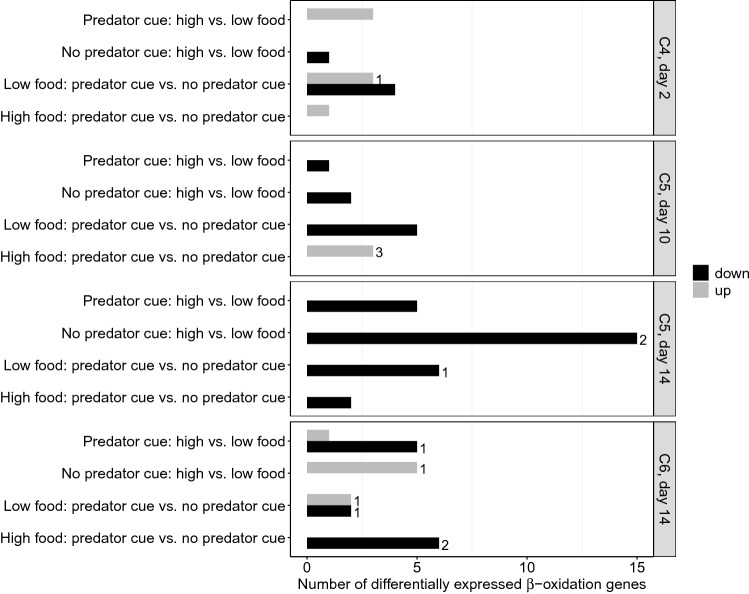
Number of significant (P < 0.05) differentially expressed ß-oxidation genes (x-axis) between experimental treatments (y-axis, left) in *Calanus finmarchicus* copepods exposed to a combination of presence or absence of a predator cue with high or low food availability. Numbers next to bars indicate numbers of genes encoding FA ligases (rate-limiting step). Comparisons of treatments were done separately per sampling day (2, 10 and 14) and stage (C4, C5 and C6, y-axis, right). The first treatment within each comparison is set as reference to the second treatment, e.g. 4 genes upregulated in “Predator cue: high vs. low food” = 4 genes up in predator cue + high food and the same 4 genes down in predator cue + low food.

**Figure 5:**
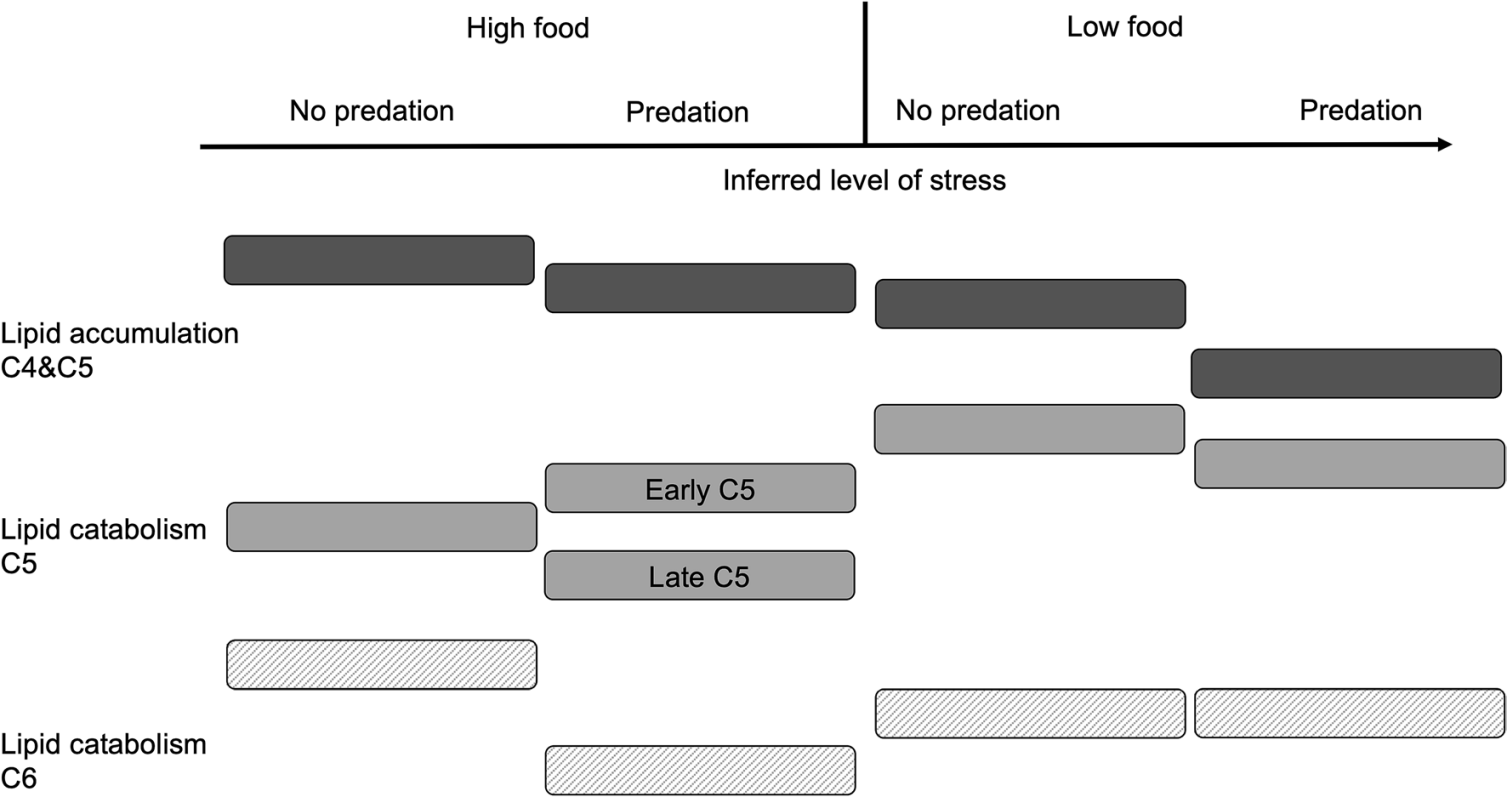
Illustration of overall lipid metabolism patterns (accumulation C4&C5: black, catabolism C5: dark grey, catabolism C6: light grey) in *Calanus finmarchicus* exposed to presence or absence of a predator cue in combination with high or low food availability while developing from C4 to C5 and further to C6. We assume that the treatment with no predator cue and high food availability represent the lowest level of percieved stress, and that the stress level increases with predator presence and low food availability. The placement of each box relative to the others are derived from the lipid fullness results and/or gene expression results. For example, lipid catabolism in C6s in the treatment with high food and no predation has more upregulated genes than the other treatments, and this box is therefore placed higher than the other treatments.

In C4s, there was no clear effect of the treatments on the expression of ß-oxidation genes, except for a pattern of upregulation in the C4s with high food and a predator cue relative to those with low food and a predator cue (Fig. 4, details presented in Supplementary Table 5). However, in early and late C5s, there was a clear pattern of differentially expressed ß-oxidation genes (Fig. 4). The first step of the ß-oxidation pathway is of particular interest because it is rate-liming^38^, which means the overall rate of the pathway is determined by the rate of that specific reaction. The reaction product is fatty acyl-CoA, and it is catalyzed by a long-chain fatty acid CoA ligase (EC number 6.2.1.3, “FA ligase” hereafter), for which several transcripts have been identified in the *C. finmarchicus* transcriptome^21^. In both early and late C5s, there was a pattern of downregulation of ß-oxidation genes with high food availability relative to low food, both with and without a predator cue (Figs. 4 and 5, details presented in Supplementary Table 5). In low food conditions, the presence of a predator cue resulted in downregulation of ß-oxidation genes relative to the group with no predator cue. In high food conditions, presence of a predator cue caused upregulation of ß-oxidation genes in early C5s relative to no predator cue (three genes, all encoding FA ligases), but downregulation in late C5s (two genes).

In C6s with a predator cue, there was a pattern of downregulation of ß-oxidation genes with high food relative to low food (Fig. 4, details presented in Supplementary Table 5). With no predator cue, there were more upregulated ß-oxidation genes with high food relative to low food. With low food availability and a predator cue there was no clear pattern in C6s, while a predator cue in combination with high food caused downregulation in C6s relative to the treatment with no predator cue present (six genes, two FA ligase genes included).

Overall lipid metabolism patterns in all treatments are summarized in Fig. 5. Our results indicate that lipid accumulation declined with predation risk, and that this decline was most prominent with low food availability. With high food availability, predation risk caused increased lipid catabolism in early C5s (based on upregulation of ß-oxidation genes). With low food availability, the predator cue caused downregulation of lipid catabolism. In late C5s, predation risk caused reduced lipid catabolism also under high food availability. In C6s, predation risk resulted in decreased lipid catabolism with high food availability, but not with low food availability.

### Diapause preparation assessment

**Figure 6:**
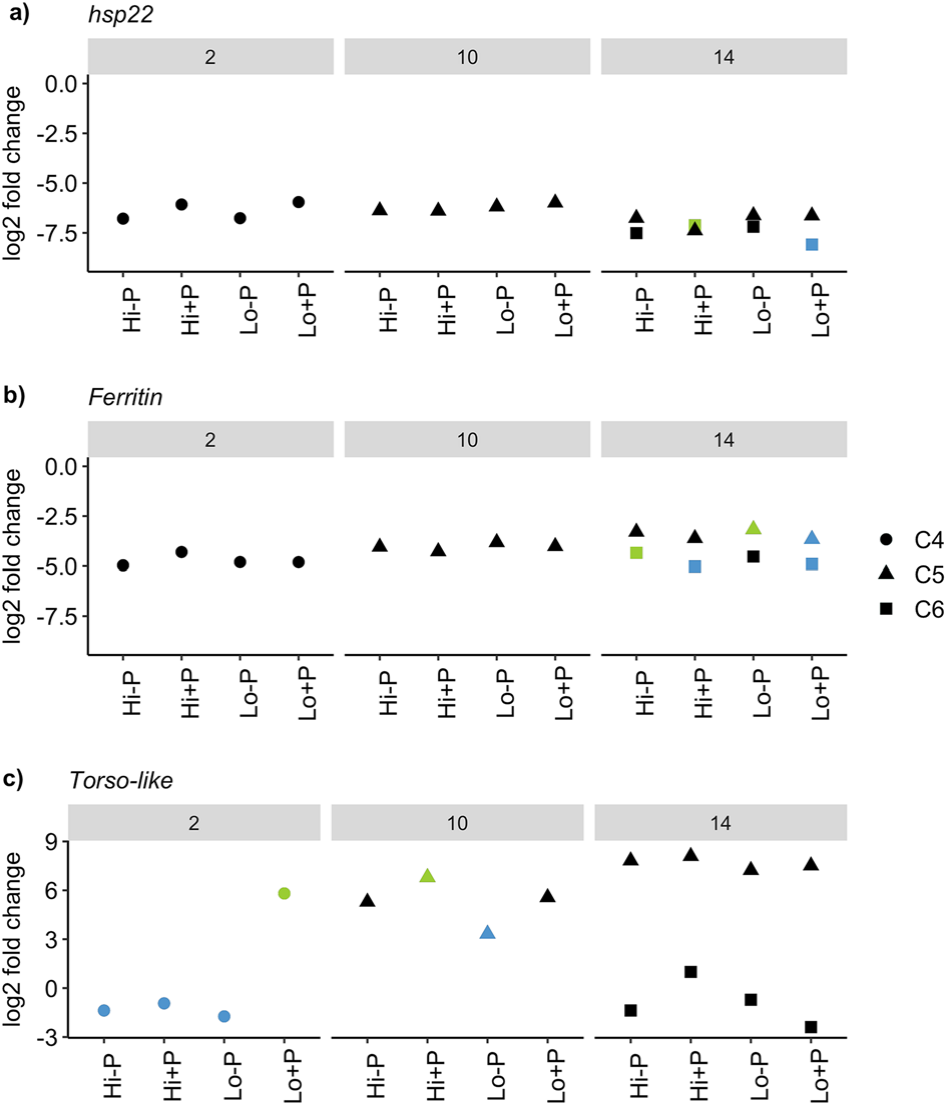
Gene expression of (**a**) *hsp22* and (**b**) *ferritin*, two molecular markers of diapause, and (**c**) *Torso-like*, a developmental marker, in *Calanus finmarchicus* copepods exposed to a combination of absence or presence of a predator cue with high or low food availability, compared to the reference group (*Calanus* spp. C5s in diapause). Within each day and stage, green symbols denote significant upregulation from blue symbols between treatments. *Hi-P *High food and no predator cue, *Hi + P *High food and predator cue, *Lo-P *Low food and no predator cue, *Lo + P *Low food and predator cue.

Expression of *hsp22* (Fig. 6a) and *ferritin* (Fig. 6b), two molecular markers showing high expression during diapause in *C. finmarchicus*^20,21,39^, was significantly (P < 0.01, details in Supplementary Table 2) downregulated in C4, C5 and C6 copepodites in all treatment groups on all days compared to the reference group. *Ferritin* was significantly upregulated in late C5s with low food and no predator compared to those with low food and a predator cue (logFC = 0.49, P = 0.049, FDR = 1), and in C6s with high food and no predator cue compared to C6s with a predator cue and high food (logFC = 0.680, P < 0.01, FDR = 0.57) and low food (logFC = 0.56, P = 0.03, FDR = 0.64, all results from statistical tests are available in Supplementary Table 2). *hsp22* was significantly upregulated in C6 with high food and a predator cue compared to those with low food and a predator cue (logFC = 0.97, P = 0.049, FDR = 1).

*Torso-like* has shown strong differentiation between early and late development within the C5 stage (i.e., expression increases as the C5s approach molting into the C6 adult stage)^26^. To our knowledge, the expression profile of this gene has not been assessed in other stages previously. There was a general pattern of low (logFC < 1) *torso-like* expression in C4s and C6s, and high (logFC > 3) expression in C5s (Fig. 6c). In C4s in the treatment with low food availability and a predator cue, *torso-like* was strongly upregulated from the reference group (logFC = 5.81, P = 0.003, FDR = 0.01, Fig. 6c, and details in Supplementary Table 1) and from the other treatments (P = 0.022 for all comparisons, details in Supplementary Table 2). *Torso-like* expression was not significantly different from the reference group in the other treatments in C4s (P > 0.05). In C5s, *torso-like* was upregulated compared to the reference group in all treatments (P < 0.05, details in Supplementary Table 1). logFC was comparable to that in the C4s low food availability and a predator cue. In C5 with high food and a predator cue, *torso-like* was upregulated relative to C5s with low food and no predator cue (logFC = 3.47, P = 0.01, FDR = 0.58). In C6, *torso-like* was neither differentially expressed from the reference group, or between treatments (P > 0.05).

A principal component analysis (PCA) was performed on the matrix of expression of all transcripts for all samples. There was a clear separation along the principal component 1 (PC1, see Supplementary Fig. 1, 35.1% variance explained), with the reference group (*Calanus* spp. C5s collected during diapause) samples grouped together to the right, followed by C4, then C5 and the C6 samples from the experiment furthest to the left. Along PC2 (23.5% variance explained), the reference group was also clearly separated from the experimental samples. There was no clear grouping of experimental treatment groups in this analysis (see Supplementary Fig. 1).

### Stage development and lipid content

**Figure 7:**
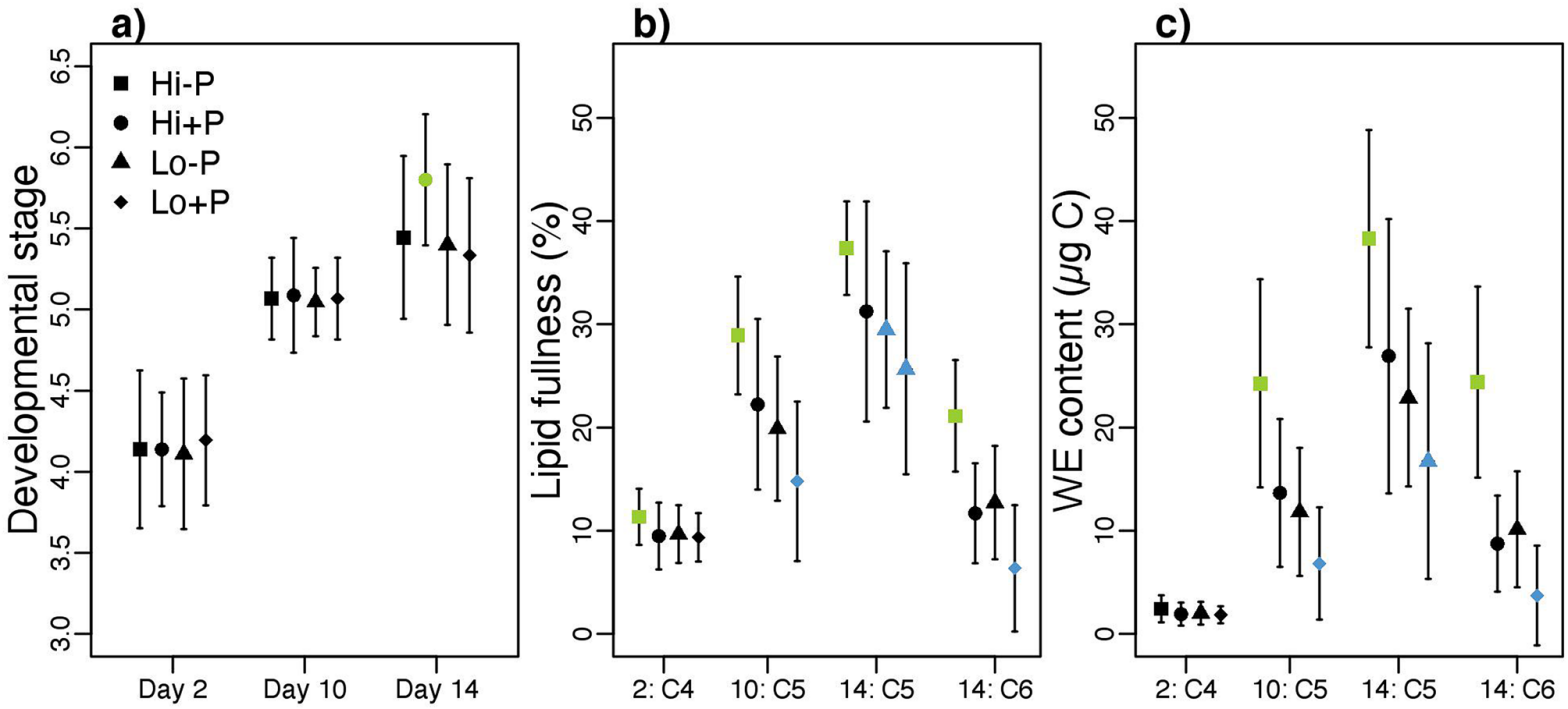
Development stage (**a**), lipid fullness (%) (**b**) and estimated wax ester (WE) content (μg C) (**c**) in *Calanus finmarchicus* copepods exposed to a combination of presence or absence of a predator cue and high or low food availability. Each treatment (see icons in legend) had three replicate tanks, each of which were sampled on each sampling day. Development stage was calculated by setting C4 = 4, C5 = 5 and C6 = 6. Symbols and vertical lines: mean values ± standard deviation (SD) per day and stage (for panels **b**, **c**).  Within each day and stage (for panels **b**, **c**), green symbols denote significantly higher estimates (P < 0.05) relative to blue symbols between treatments. *Hi-P *High food and no predator cue, *Hi + P *High food and predator cue, *Lo-P *Low food and no predator cue, *Lo + P *Low food and predator cue.

Here, we present basic physiological parameters (stage development, lipid fullness and estimated WE content) of the copepods sampled for RNA seq on days 2, 10 and 14. Data on stage development and lipid fullness from a larger pool of copepods from the same experiment are examined in more detail in our recent study^33^. At the termination of the experiment (day 24) 99% of the remaining copepods had reached the C6 stage. We calculated the mean development stage of the copepods sampled for RNAseq per sampling day (days 2, 10 and 14) by setting C4 = 4, C5 = 5 and C6 = 6. The mean developmental stage across treatments was 4.1 on day 2, 5.1 on day 10 and 5.5 on day 14. There were no significant differences in developmental stage distribution between treatments on days 2 or 10, while on day 14, the development stage distribution was higher (i.e. more advanced development) with high food and predator cue compared to the other treatments (Fig. 7a, P-values from Wilcoxon rank sum tests of differences between treatments < 0.05, all results from statistical test are available in Supplementary Table 6).

In general across treatments, lipid fullness increased from C4 to C5 and was highest in late C5s (Fig. 7b, P < 0.05 in all comparisons, all results from statistical test are available in Supplementary Table 6). Lipid fullness was lower in C6s (females only included in analysis) than in C5s, but generally higher in C6s than in C4s (P < 0.05). Comparing treatments, lipid fullness in C4s was significantly higher with high food and no predator cue (P-values from Wilcoxon rank sum tests of differences to other treatments = 0.02 for all comparisons). In early C5s (day 10), lipid fullness was also highest in those with high food and no predator cue (P < 0.05 in all comparisons), intermediate and statistically indistinguishable from each other with high food and predator cue/low food and no predator cue (P = 0.15), and lowest with low food and predator cue (P < 0.05 in all comparisons). Patterns of lipid fullness for late C5s (day 14) and C6s resembled early C5s, except that for the late C5s, differences were only statistically significant (P < 0.05) between high food without predator cue and the low-food treatments (for the other combinations, P = [0.11–0.17]).

Estimated wax ester (WE) content (Fig. 7c) was always found to be below the suggested threshold for diapause entry of 70 μg C^22^ (maximum value = 58.3 in C5 day 14 with high food and no predator cue). WE content was not significantly different between treatments for C4 (P > 0.05), while for C5 (early and late) and C6 the significant differences were similar to the pattern for lipid fullness described above for C6 and early C5s (all results from statistical test are available in Supplementary Table 6).

### Species identification

The species identity in both the maternal generation (n = 121) of the copepods used for the experimental treatments included in the present study, as well as a subsample of other offspring from this generation (n = 74), were confirmed by genetic markers to be exclusively *C. finmarchicus*. The species identification analyses were performed to rule out the possibility of having other *Calanus* species present in the culture together with *C. finmarchicus*. It was indeed recently reported that both *C. finmarchicus* and *C. glacialis* occur in the Trondheimsfjord^3^, from where the NTNU Sealab culture was originally collected (in 2004).

## Discussion

We have demonstrated that predator cues and food availability interactively influence lipid metabolism in *C. finmarchicus* copepodites developing from the C4 copepodite stage via C5 to the adult female stage. Lipid metabolism varied between treatments, life stages and sampling day. Interestingly, lipid catabolism was upregulated in response to perceived predation risk in early C5s with high food conditions but downregulated in late C5s. With low food conditions, lipid catabolism was downregulated in both early and late C5s in the presence of a predator cue. This suggest that food availability modulates the lipid metabolism response to predator presence, and that the response changed with time. In addition, our results indicate that lipid accumulation was disrupted by predator presence, particularly in low food conditions. This illustrates that lipid metabolism in *C. finmarchicus* is a dynamic process which is in part influenced by the interacting environmental effects of predation pressure and food availability. The copepods did not enter diapause in this experiment. However, as lipid accumulation is important in controlling diapause in *C. finmarchicus*^25^, our findings can contribute to understanding more of the metabolic processes preceding or leading to diapause in *Calanus* copepods.

Independently of treatment, lipid fullness increased during the development from C4 to early C5 and late C5, before it declined in C6. These results are consistent with previous observations^26,33^ and illustrates that the C4s and C5s store lipids in preparation for either diapause or for the final molting into the reproductive C6 stage. In both early and late C5s, there was a pattern of downregulation of ß-oxidation genes with high food availability relative to low food, both with and without a predator cue. These results imply that high food availability results in lower lipid catabolism than low food availability, which can be expected in favorable conditions. C4s and C5s with high food availability and no predator cue, and presumably the lowest level of stress, accumulated more lipids from early on in the experiment (day 2) compared to other treatments. The observed downregulation of several ß-oxidation genes in the copepodites in this treatment indicates a lower lipid catabolism rate, and illustrates that these copepodites had a minimal need to utilize lipids for energy. This may be explained by a lower need to physiologically compensate for stress compared to copepods in the other treatments.

The response to predation risk differed within the food availability treatments. When a predator cue was present in the treatment with high food, we observed increased lipid catabolism in early C5s, which changed to downregulation in late C5s. In the natural environment of copepods, predators are omnipresent, though the degree of predation risk varies depending on e.g. depth, season and time of the day. Copepods are known to respond to chemical cues from predators by altering their life history strategies, e.g. growth or reproductive rates^13,14^. In our controlled experiment, high concentrations of chemical cues from a predator preying upon *C. finmarchicus* may be perceived as a stressor, at least when compared to the copepods in filtered seawater. Comparable upregulated lipid catabolism responses to (chemical) stressors have been observed in other crustaceans such as *Machrobrachium borellii*^28,40^ and *Homarus americanus*^30^. Alternatively to the response being stress-related, the upregulation of ß-oxidation genes in early C5s in the treatment with high food and a predation cue may be due to a higher energy demand in these copepodites while preparing to molt to the adult stage, as corroborated by the accelerated developmental progression in this treatment (day 14). On day 14, close to the terminal molt, we observed downregulation of ß-oxidation genes as a response to predation risk, with both low and high food availability. This illustrates that lipid metabolism is a dynamic process that changes during development. Our observations of general ß-oxidation gene expression pattern across treatments, relative to the reference group, further supports a higher lipid catabolism rate in C4s and early C5s, and a decline in late C5s and C6s. In a recent study by Skottene et al*.* (2019), where *Calanus* C5s terminated diapause without available food, lipid catabolism was also higher early in the C5 stage and declined close to the terminal molt to C6^21^. Thus, a similar pattern in lipid catabolism seems to occur in the late life stages in *Calanus* copepods, perhaps regardless of activity level, but modulated by both food availability and predation risk*.* Exposure to oil pollution has been observed to disrupt lipid catabolism^32^, indicating additional sensitivity to anthropogenic disturbances. In *Neocalanus flemingeri* lipid catabolism genes were in contrast upregulated towards the end of diapause (which occurs in adults), and did not become reduced^41^. This underlines species differences and the complexity of energetic metabolism in diapausing zooplankton species.

With low food availability, predator presence resulted in downregulation of ß-oxidation genes in both early and late C5s. This downregulation of lipid catabolism genes in the presence of a predator cue could indicate that the copepods may cope with stress by reducing their overall metabolism. Downregulated lipid metabolism in response to petroleum oil components was recently reported in *C. finmarchicus*^32^, and predation risk has been linked to lower resting metabolic rates in the amphipod *Gammarus minus*^42^, and to decreased swimming speed in the marine copepod *Temora longicornis*^43^. Reduced activity, causing a reduced energy demand, is a common response to predation risk in aquatic and terrestrial prey^44^, and may explain the decreased lipid utilization in the copepods exposed to a predator cue. Specifically, diel vertical migration to deeper waters with lower food availability, where metabolism is generally reduced^45,46^, is a common predator avoidance behavior in zooplankton^47–49^. Though not feasible to assess in the relatively small experimental tanks in this study, it is possible that the lower lipid metabolism rate that we observed resulted from a general reduction in activity in response to predation risk.

The copepods with low food availability and a predator cue accumulated lower amounts of storage lipids than copepods in the other treatments. This was reflected by the downregulation of the lipid storage-associated gene *FABP* in this treatment in early C5s (and C6s). Interestingly, *FABP* and *ELOV* showed consistently upregulated expression (despite high FDRs: 0.65-1) in the early and/or late C5s with no predator cue and low food availability. This suggests that these copepodites had the highest lipid biosynthesis of the treatments, or at least that the copepodites were metabolically primed to synthesize lipid from dietary substrates as they became available. Our results imply that under low food conditions, copepodites will prioritize accumulating lipid stores over other metabolic demands, but the presence of a predator cue seems to disrupt lipid accumulation. As discussed in the previous paragraph, the copepods may respond to predator presence as a stressor, and reduce their overall lipid metabolism, i.e. both catabolism and accumulation.

When adulthood is reached, female *C. finmarchicus* use a combination of endogenously stored lipids and feeding to fuel egg production^50,51^. This is reflected in the general reduction in lipid fullness in C6 compared to C5s. As in C4s and C5s, lipid fullness in C6s was highest in copepods with high food conditions and no predator cue, and lowest in those with low food and a predator cue. However, the gene expression patterns in the C6s differed, to some extent, from those in the C5s. Under high food conditions, lipid catabolism in C6s was reduced in the presence of the predator cue, like in late C5s. This could be linked to reduced energy demands caused by e.g. reduced movement, but this was not quantified. Under low food conditions, the presence of a predator cue had no clear effect on lipid catabolism (no DEGs). This contrasts with the clear downregulation of lipid catabolism observed in the C5s with low food conditions. Differences in lipid catabolism responses between life stages have been observed in *Calanus* copepods exposed to petrogenic oil components^32^. It is likely that egg production rates differed between the treatments, as both food availability and predation risk can affect reproduction in copepods^14,52–54^. As reproduction and lipid metabolism rates are likely closely linked, egg production should be quantified and included in future studies evaluating lipid metabolism rates in adult copepods.

Regarding lipid metabolism in general, our findings show that food availability influences the lipid metabolism response to predation risk in *C. finmarchicus* (Fig. 5), evident by the response to predation being different depending on food availability*.* During their life cycle, *Calanus* spp. experience periods when food availability is too low to meet metabolic demands^55,56^. The length and intensity of these periods are likely to change with global warming, which can impact the *Calanus* spp. life cycle by causing spatial and temporal mismatches between copepod populations and phytoplankton blooms^57,58^. Changes in predation pressure are also likely^59^. Our results indicate that lipid metabolism is a sensitive endpoint for changes in food availability and predation pressure. As lipid content and/or composition likely is closely linked to the initiation and termination of diapause^23,60^, anthropogenic and environmental factors that change lipid metabolism can indirectly alter the timing of diapause. A change in diapause timing could further exacerbate trophic mismatches caused by changes in ocean temperature.

Our assessments of physiological, developmental and transcriptomic parameters strongly imply that none of the experimental copepods began diapause preparation. No C5s from any treatments achieved a lipid level close to the proposed threshold *C. finmarchicus* C5s need to support the energetic costs related to diapause (70 μg C wax esters^22^). Interestingly, there were indications of slightly higher expression of the diapause-associated gene *ferritin* in late C5s and C6s without a predator cue. Though the upregulation of diapause-associated genes in C6 is difficult to interpret, the upregulation in late C5s without a predator cue and low food availability is consistent with diapause preparation. However, due to the large difference in differential expression from the reference group of copepods in diapause (> logFC −2.5), and the small difference in gene expression between the two treatments (> logFC 0.7), it is more likely that this upregulation of *ferritin* is related to other activities than diapause preparation. The protein ferritin is for instance known to have roles in iron binding in the copepod *Artemia fransiscana*^61^.

At the end of the experiment, almost all of the copepodites had molted into adults, and molting occurred faster in tanks with high food and predator cue compared to other treatments (see also Kvile et al*.* for more details regarding stage progression^33^). The upregulation of *torso-like* in early C5s in this treatment further supports this. Meanwhile, the strong upregulation of *torso-like* in C4s with a predator cue but with low food availability, may indicate faster development on a molecular level, though sufficient food may be essential for actual molting to occur. Similar faster developmental rates under predation risk have been reported in *T. longicornis*, of which late nauplii stages showed increased molting probability in the presence of fish kairomones^62^.

Perceived predation risk may be an important environmental cue that determine diapause initiation in the field, but our results supports previous observations^8,10^ that other cues are likely required either in addition, or instead of, predation risk. Additional potential explanations why the copepods did not exhibit clear signs of diapause preparation in this study include: (i) predation risk does not induce diapause either alone or in combination with other cues; (ii) the predator cue triggering diapause is not chemical (but rather e.g. hydrodynamic or visual); or (iii) the lab conditions are too far from the natural environment, and that these conditions over several generations have favored continuous development. Although the 3rd point is difficult to overcome, it would be of interest to run experiments including other potential diapause cues such as temperature, or increased pressure to simulate a deep-water environment, or to assess the response to chemical predator cues in field-collected *C. finmarchicus*.

In conclusion, our results demonstrate that lipid metabolism is sensitive to variations in food availability and perceived predation risk. Interestingly, the lipid metabolism response to predator presence in *C. finmarchicus* differed depending on the food availability, and with time and between life stages. Our molecular analyses showed no evidence of diapause preparation in our experiment, though there were some indications of altered gene expression of diapause and developmental markers depending on food availability and the presence of predation. Because diapause induction, duration and termination may be directly related to lipid content^22–24^, and our observations show that both food availability and predation risk affects lipid metabolism, both factors can indirectly influence diapause timing. A change in diapause timing can alter entire ecosystem dynamics, which are already under threat by anthropogenic disturbances like climate change and pollution.

## Methods

### Copepod collection and species determination of the culture

The reference group, consisting of *Calanus* spp. copepodites of stage C5, was collected from sea bed depth (400 m) up to 200 m in the Trondheimsfjord, Norway (N63° 29′, E10° 18) in August 2017 using methods described in Skottene et al*.*^21^. The copepod samples (n = 3, 10 individuals per tube) were placed in microcentrifuge tubes with 1.5 mL of RNAlater (ThermoFisher, USA) as soon as possible after collection, while onboard the research vessel. All disturbance was minimized as far as possible, for details see the Methods section in Skottene et al*.* (2019). For the experiment, copepods from the continuous *C. finmarchicus* culture at the NTNU Sealab facility in Trondheim, Norway, were used. The culture was started with individuals collected by vertical net-hauls at a station in the Trondheimsfjord in the autumn of 2004. At the time, it was assumed that *C. finmarchicus* was the only species of *Calanus* in the area, and confirmation of species were based solely on morphological criteria. However, it was recently documented that *C. glacialis* is present together with *C. finmarchicus* in the Trondheimsfjord^3^, and that the two species can only be reliably distinguished using molecular tools^63^. Therefore, we used genetic markers to verify the actual species composition of the *Calanus* culture. For this purpose, 200 females were sampled randomly from the culture, representing two generations: (1) the maternal generation of the copepods used in the experiment, and (2) a subsample of offspring from this maternal generation, i.e. the same generation as the copepods used in the experiment. The females were anesthetized with MS-222 (Finquel, 1.5 g L^−1^ seawater, Argent Labs, USA), imaged for reference by a ccd camera (DS Fi1/U2, Nikon Inc., Japan) attached to a dissecting microscope (MZAPO, Leica Microsystems, Germany). After imaging, the individuals were preserved separately in 70% ethanol at room temperature, transferred to storage at + 4 °C until transport to the laboratory and final storage at − 20 °C. Later, species identification of each specimen was performed following the protocol described in Choquet et al*.* (2017). In short, DNA was extracted from the two antennules of each individual using a HotShot-based protocol. This DNA was then used as a template for the amplification of six molecular markers type InDel^64^, multiplexed in one PCR reaction per individual. The resulting amplified fragments were sized using a 3500xL Genetic Analyzer (Applied Biosystems, USA) to establish the genotype of each individual (n = 194 with successful amplification) and determine the species.

Species identification analysis of the reference group samples was not performed. The RNA extraction for RNA seq analysis of the copepods in the reference group samples was done before the new data about *Calanus* species composition in the Trondheimsfjord^3^ was available. However, as *C. finmarchicus* is the dominant species in the Trondheimsfjord^3^, and because the two species show close similarity in diapause behavior^6^, we can assume that the reference group is representative of *C. finmarchicus* C5 copepodites in a state of early diapause and very low metabolism. Gene expression analyses of several of the same target genes did not show any clear influence of the species composition in a previous study^21^.

### Experimental setup

On day 0 of the experiment, in total 3600 C4 copepodites were transferred from the culture to 12 experimental tanks (300 copepodites per 45 L white polyethlene containers with lid, three replicates per treatment, Fig. 1). We used a 2 × 2 factorial design with three replicates in each treatment: high food and no predator cue (Hi-P), high food and predator cue (Hi + P), low food and no predator cue (Lo-P), low food and predator cue (Lo + P) (Fig. 1). Aliquots of 50 copepods were sorted and assigned randomly to tank until reaching 300 per tank. Specifically, copepods were picked up with plastic spoons and kept submerged while quickly determining stage from visual inspection of size. The tanks received daily natural filtered (10 µm mesh) seawater collected at 70 m depth in the Trondheimsfjord at an exchange rate of 1.5 times the tanks’ volume. The temperature was kept constant within 10 ± 2 °C and a light–dark cycle 16:8 h, corresponding to the conditions of the culture. Photoperiod is not considered a trigger for diapause induction^65,66^, and was therefore not an assessed parameter in the present study. From day 1, the water was enriched with the unicellular algae *Rhodomonas baltica* at concentrations resulting in 200 (high food) or 90 (low food) μg C/L. These levels are above (high food) and below (low food) the threshold at which the response of *C. finmarchicus* development rate to food level flattens out (see Fig. 6B in Campbell et al*.*^67^). Additionally, the tanks received water with predator cue (+ P) or regular filtered water (−P) at a rate replacing 10% of the tanks’ volume daily. The filtered seawater is unlikely to contain significant concentrations of predator cues as these are quickly degraded^68^. Water with predator cues was obtained by continuously pumping seawater through a 20 μm filter from a separate 37 L tank with lumpfish (*Cyclopterus lumpus*) juveniles. Lumpfish are opportunistic feeders on a range of zooplankton species in the wild, including *C. finmarchicus*^69,70^, and the lumpfish juveniles in our experiment readily preyed upon the offered copepods. Since *Daphnia* respond more strongly to chemical signals from a predator preying on conspecifics than to the predator of crushed conspecifics alone^71^, we fed the lumpfish live *C. finmarchicus* from the main culture (20–50 stage C5 or C6 per fish daily, spread out in at least four meals) to ensure a continuous predation signal. The predator cue in this study is therefore potentially a combination of kairomones from the fish and alarm signals from copepods eaten by the fish. We started with 114 fish with a mean weight of 0.34 g. This was reduced to 54 on day 12 to account for an assumed doubling of the fish weight (O. A. Kjørsvik, personal communication; mean weight of removed fish 0.73 g). The experiment was terminated on day 24, at which point the remaining fish had a mean weight of 1.22 g and were euthanized using an overdose of tricaine methanesulfonate solution (Finquel, Argent Laboratories, Redmond, WA, US).

We sampled copepods for RNA seq from all tanks in random order on days 2, 10 and 14. We randomly collected 12 (day 2) or 15 (days 10 and 14) copepods per tank using a ladle, keeping the samples (copepods + tank water in a plastic cup) cooled on ice. Copepods were then anesthetized with tricaine methanesulfonate (1.5 g L^−1^ seawater), identified to stage and photographed laterally with using a CCD camera (model DS-Fi1/U2) mounted on a Leica MZAPO stereo microscope (Leica Microsystems, Wetzlar, Germany). After this, copepods were transferred to 2 mL Eppendorf tubes with RNAlater (ThermoFisher, Waltham, USA) and kept at 4 °C for 24 h and then at − 20 °C until analyses. To minimize handling time, we anesthetized and photographed ~ 5 copepods at a time.

We used the photographs to determine lipid fullness, i.e. the percentage of the prosome area comprised by the lipid sac area^72^. We quantified prosome and lipid sac area by manually outlining these features using the free software ImageJ^73^ and a drawing tablet (Wacom Cintiq 12wx, Wacom Co., Ltd., Saitama, Japan), calibrating the pixel-to-mm ratio using an image of a calibration stage micrometer. To avoid the additional uncertainties in converting from area to volume^72^, we defined lipid fullness as the percentage of the body area comprised by the lipid sac area (100 × lipid area/body area).

To assess the WE content in relation to the proposed threshold by Rey-Rassat et al. (2002), WE content (µg C ind.^−1^) was calculated as:

1 $${\text{WE }} = \, \left( {{1}000 \times {\text{lipid volume }}\left( {{\text{mm}}^{{3}} } \right) \times 0.{86} \times 0.{78}} \right)/{1}.{44}$$

We calculated lipid sac volume using the equation^74,75^:

2 $${\text{V }} = \, \left( {\pi {\text{A}}^{{2}} } \right)/{\text{4L}}$$

where A = area and L = length of major axis (lipid or prosome length). To quantify effects of treatments on development, we compared the distribution in developmental stages per sampling day and tank (setting C4 = 4, C5 = 5, C6F/C6M = 6). Preliminary analyses using parametric statistical tests indicated that the assumption of normality of the residuals was not always met (Shapiro Wilk test, P < 0.05). Therefore, significant differences between treatments in development stage per day were tested using the nonparametric Kruskal–Wallis one-way ANOVA and, subsequently, the two-sided Wilcoxon rank sum test to compare pairs of treatments. Similarly, we tested for significant differences in lipid fullness and WE content between treatments per day and stage. All details from the tests are available in Supplementary Table 6.

### RNA isolation, library preparation and RNA seq

On day 2, 80% of sampled individuals were at stage C4 (with 3% C3 and 17% C5), while on day 10, 92% of sampled individuals were at stage C5 (with 1% C4, 6% C6 females and 2% C6 males). We therefore focused the RNA seq analyses exclusively on stage C4 for day 2, and on C5 for day 10. On day 14, 51% of sampled individuals were stage C5, 40% C6F and 9% C6M. Thus, we analyzed samples of both stage C5 and C6F from each tank on day 14 (see Supplementary Table 7 for details).

We pooled copepods of the same stage from the same tank and day (n = 3–15 individuals, mean: 9, see Supplementary Table 7 for n in each sample) in order to incorporate biological material from a larger number of individuals into a limited number of RNA-seq libraries^76^. This is common in gene expression studies with copepods^77^. On day 2, one sample per treatment was analyzed as we did not expect one day of differential treatment to affect transcription. On days 10 and 14, three samples per treatment were analyzed. RNA extraction from copepod samples was performed using the Qiagen RNeasy Plus Universal Mini Kit (Qiagen Inc., Valencia, CA, USA) with the additional use of a QiaShredder column, following the manufacturer’s protocol. RNA quality assessment was performed using a Model 2100 Bioanalyzer instrument (Agilent, Santa Clara, USA) using methods described in previous studies^21,32^. All the analyzed samples were of high quality, containing a strong 18S band and little or no evidence of genomic DNA contamination (large bands) or degradation (smear of smaller bands).

For RNA sequencing, cDNA libraries were synthesized from total RNA (80 ng/mL RNA input) using the Illumina TruSeq Stranded mRNA sample preparation kit (Illumina, San Diego, USA). Final volume of cDNA libraries was 22 µL. Prior to RNA sequencing, the cDNA libraries were pooled and normalized, and a quality control was performed on a Bioanalyzer instrument by the sequencing facility.

### Illumina sequencing and bioinformatic analyses

The 43 samples were sequenced at the Genomic Core Facility (GCF) at NTNU, Trondheim, The libraries were clustered on a Nextseq500 high output flowcell, and 75 bp paired-end sequencing was performed on a NextSeq500 instrument (Illumina inc., San Diego, CA, USA) according to the manufacturer’s instructions. One library (C6, day 14, low food, no predation) failed sequencing, leaving 42 samples. Generated sequences were demultiplexed and adapter trimmed at the GCF. All samples passed standard fastq (FastQC software, version 0.11.8) quality checks, with ~ 500 million reads retained in total, and ~ 12 million reads per sample (Supplementary Table 7).

Read mapping and estimation of abundance were performed using scripts bundled within Trinity (version 2.8.4)^78^. Reads were mapped to the a previously assembled^26^ and newly annotated^37^ transcriptome (PRNJA231164,) using Bowtie (version 2.3.4.2)^79^. Read counts were normalized to trimmed mean of M-values (TMM) to account for differences in library size, and FPKM-normalized (fragments per feature kilobase per million reads mapped) when producing the PCA-plot, using scripts bundled within Trinity. Transcript abundances were estimated using the RSEM package^80^ (version 1.3.2).

Differentially expressed genes (DEGs, P < 0.05) were determined using edgeR (version 3.28.0) and limma (version 3.42.0) within Bioconductor^81^ in R (version 3.6.0). DEGs were identified using a generalized linear model (GLM) fitted with quasi-likelihood F-tests. We compared (i) the reference group with the different treatments each sampling day, and (ii) the treatments with each other within each sampling day (stages analyzed separately: C4 on day 2, C5 on day 10 and C5 and C6F on day 14). Stage (C4, C5 and C6), sampling day (2, 10 and 14) and treatment (high or low food,  predator cue or no predator cue) were included as factors in the design matrix in the GLM. Expected gene counts from RSEM were used as input, and genes with very low counts per million (CPM < 1) were filtered out. Tagwise dispersion was calculated using the Cox-Reid profile-adjusted likelihood method, which allows for multiple factors in the GLM^81,82^. Gene expression results are given on log2 scale, alpha level was set to 0.05.

We selected specific target genes (Table 1) for assessing differential expression, primarily based on their biological functions in *Calanus* copepods, and on their expression patterns in previous studies. The effect of time (days since start of experiment) on the total number of up and downregulated ß-oxidation DEGs, was assessed using two separate linear models in R (version 3.6.2). The residuals of both models showed a normalized distribution, and Shapiro tests on residuals of both models confirmed this distribution (P > 0.05).

## Supplementary information

Supplementary Information.

Supplementary Table 1.

Supplementary Table 2.

Supplementary Table 3.

Supplementary Table 4.

Supplementary Table 5.

Supplementary Table 6.

Supplementary Table 7.

## Data Availability

Sequence data have been submitted to the National Center of Biotechnology Information (NCBI; www.ncbi.nlm.nih.gov) under the Bioproject PRJNA593934. Genotypes of InDels are submitted to DRYAD, 10.5061/dryad.1rn8pk0qp^83^.
