# Supplementary material for: Lipid metabolism in Calanus finmarchicus is sensitive to variations in predation risk and food availability
Source: Sci Rep. 2020 Dec 18;10:22322. doi: 10.1038/s41598-020-79165-6 (PMC7749129; doi:10.1038/s41598-020-79165-6)
Supplement: Supplementary file 1 — Supplementary Information. [file 41598_2020_79165_MOESM1_ESM.docx]

**Lipid metabolism in *Calanus finmarchicus* is sensitive to variations in predation risk and food availability**

Elise Skottene^1*^, Ann M. Tarrant^2^, Dag Altin^1,3^, Rolf Erik Olsen^1^, Marvin Choquet^4^ and Kristina Ø. Kvile*^2,6^

^1^Department of Biology, Norwegian University of Science and Technology, Trondheim, Norway

^2^Department of Biology, Woods Hole Oceanographic Institution, Woods Hole, Massachusetts, USA

^3^BioTrix, Trondheim, Norway

^4^Faculty of Biosciences and Aquaculture, Nord University, Bodø, Norway

^5^Centre for Ecological and Evolutionary Synthesis (CEES), Department of Biosciences, University of Oslo, Oslo, Norway

^6^Norwegian Institute for Water Research, Gaustadalléen 21, 0349 Oslo, Norway

*corresponding author: [elise.skottene@ntnu.no](mailto:elise.skottene@ntnu.no)

*corresponding author: [k.o.kvile@ibv.uio.no](mailto:k.o.kvile@ibv.uio.no)

Supplementary Table 1. Gene expression results of *ELOV* (elongation of very long chain fatty acids), *FABP* (a fatty acid binding protein), *ferritin*, *hsp22* and *torso-like* in *C. finmarchicus* copepods exposed to a combination of a predator cue and high or low food availability, compared to the reference group (*Calanus* spp. C5s in diapause). Hi-P=High food and no predator cue; Hi+P=High food and predator cue; Lo-P=Low food and no predator cue; Lo+P=Low food and predator cue. logCPM: log2 counts per million. FDR: false discovery rate. (Submitted as Excel file).

Supplementary Table 2. Gene expression of *ELOV* (elongation of very long chain fatty acids), *FABP* (a fatty acid binding protein), *ferritin*, *hsp22* and *torso-like* in *C. finmarchicus* copepods exposed to a combination of a predator cue and high or low food availability. The first group within each comparison is set as reference to the second group in each comparison, e.g. 4 genes upregulated in Lo+P vs Lo-P = 4 genes up in Lo+P and the same 4 genes down in Lo-P. Hi-P=High food and no predator cue; Hi+P=High food and predator cue; Lo-P=Low food and no predator cue; Lo+P=Low food and predator cue. logCPM: log2 counts per million. FDR: false discovery rate. (Submitted as Excel file).

Supplementary Table 3. Gene expression of lipid metabolism genes (P<0.05) previously identified by Lenz *et al.* (2014) and Tarrant *et al.* (2016) (desaturases, fatty acid synthetases, elongases and phospholipid acyltransferases) in *C. finmarchicus* copepods exposed to a combination of a predator cue and high or low food availability, compared to the reference group (*Calanus* spp. C5s in diapause). Hi-P=High food and no predator cue; Hi+P=High food and predator cue; Lo-P=Low food and no predator cue; Lo+P=Low food and predator cue. logCPM: log2 counts per million. FDR: false discovery rate. Note that the file contains one tab per stage/day. (Submitted as Excel file).

Supplementary Table 4. Gene expression of ß-oxidation genes (P<0.05) previously identified by Skottene *et al.* (2019) in *C. finmarchicus* copepods exposed to a combination of a predator cue and high or low food availability, compared to the reference group (C5s in diapause). Hi-P=High food and no predator cue; Hi+P=High food and predator cue; Lo-P=Low food and no predator cue; Lo+P=Low food and predator cue. logCPM: log2 counts per million. FDR: false discovery rate. (Submitted as Excel file).

Supplementary Table 5. Gene expression of ß-oxidation genes (P<0.05) previously identified by Skottene *et al.* (2019) in *C. finmarchicus* copepods exposed to a combination of a predator cue and high or low food availability. The first group within each comparison is set as reference to the second group in each comparison, e.g. 4 genes upregulated in Lo+P vs Lo-P = 4 genes up in Lo+P and the same 4 genes down in Lo-P. Hi-P=High food and no predator cue; Hi+P=High food and predator cue; Lo-P=Low food and no predator cue; Lo+P=Low food and predator cue. logCPM: log2 counts per million. FDR: false discovery rate. (Submitted as Excel file).

Supplementary Table 6. Results from the non-parametric two-sided Wilcoxon rank sum test of differences between treatments or stages. The file contains one tab per analysis: 1) Development stage per day and treatment; 2) Lipid fullness per day and stage; 3) Estimated wax ester (WE) content per day and stage; 4) Lipid fullness per day/stage and treatment; 5) WE per day/stage and treatment. In each table, the first row shows the mean values per stage, day and/or treatment (indicated by column). The lower rows show the test statistic (W) and P-value from the Wilcoxon rank sum test of differences between the group indicated in the column and group indicated in the row. Hi-P=High food and no predator cue; Hi+P=High food and predator cue; Lo-P=Low food and no predator cue; Lo+P=Low food and predator cue. (Submitted as Excel file).

Supplementary Table 7. Accession numbers and number of reads per library of *Calanus finmarchicus.* (Submitted as Excel file).

Supplementary Figure 1. Principal Component Analysis (PCA) plot of gene expression in all *Calanus finmarchicus* samples. Colors indicate different treatments, symbol shape indicates stage. The reference group (C5s in diapause) clustered together to the lower right, C6 (day 14) clustered to the middle let. C4s (day 2) were closer to the reference group than the C5s (days 10 and 14), which clustered between C4s and C6s. There was no clear pattern among treatment groups in the PCA. Hi-P=High food and no predator cue; Hi+P=High food and predator cue; Lo-P=Low food and no predator cue; Lo+P=Low food and predator cue.
